# 

**Published:** 1903-10-24

**Authors:** 


					u?ti . "The Standard of highest purity."?Lancet. October 24th, 1903.
CADBURYS pit U CADBURYS
nnnna ?\ JITJlL IMr& onnnn
COCOA I, M, M JE? . . COCOA
'A PERFECT FOOD." HO DRUOS OR ALKALIES UIXD.
fiNo. 891. 'Vol. XXXV. |JS5S?1 Saturday,
Oct. 24th, 1903.
[SMbMri^ionTenHB,] pR|CE THREEPENCE.

				

